# Quality of Life, Depression, Anxiety, and Stress in Humanities Students and People living with HIV in Russia during the COVID-19 Second Wave

**DOI:** 10.1192/j.eurpsy.2025.1867

**Published:** 2025-08-26

**Authors:** V. I. Rozhdestvenskiy, V. V. Titova, I. A. Gorkovaya, Y. S. Aleksandrovich, D. O. Ivanov

**Affiliations:** 1 Department of Psychosomatics and Psychotherapy, Saint Petersburg State Pediatric Medical University, Saint Petersburg, Russian Federation

## Abstract

**Introduction:**

During the pandemic of a new coronavirus infection in Russia, the number of patients with diagnosed depressive and anxiety disorders increased. Studies have shown a sharp increase in the number of respondents who recorded stress as a non-specific reaction to a sudden change in environmental conditions. Quality of life, encompassing physical, psychological, and social well-being, often correlates with individuals’ emotional and mental states.

**Objectives:**

The study aimed to examine the associations between quality of life and emotional states — specifically, depression, anxiety, and stress — in humanities students and people living with HIV.

**Methods:**

Data were collected from January to July 2021 through a Google form. The sample comprised 35 humanities students from Russian universities and 59 HIV-positive patients. The WHOQOL-BREF assessed quality of life, while the DASS-21 measured levels of depression, anxiety, and stress. Both questionnaires have been adapted for Russian respondents.

**Results:**

We found that in the student group, depression was related to physical and psychological well-being (r_s_ = -0.491, p < 0.01), self-image (r_s_ = -0.552, p < 0.05) and microsocial support (r_s_ = -0.550, p < 0.05), and anxiety with physical and psychological well-being (r_s_ = -0.356, p < 0.05) and microsocial support (r_s_ = -0.353, p < 0.05). In the patient group, physical and psychological well-being was associated with depression (r_s_ = -0.309, p < 0.05) and anxiety (r_s_ = -0.269, p < 0.05); self-perception was associated with depression (r_s_ = -0.490, p < 0.01), anxiety (r_s_ = -0.311, p < 0.05) and stress (r_s_ = -0.361, p < 0.05); microsocial support — with depression (r_s_ = -0.381, p < 0.01), anxiety (r_s_ = -0.260, p < 0.05) and stress (r_s_ = -0.322, p < 0.05); social well-being — with depression (r_s_ = -0.360, p < 0.01), anxiety (r_s_ = -0.426, p < 0.01) and stress (r_s_ = -0.334, p < 0.05).

**Conclusions:**

The study revealed distinct patterns in the relationship between life quality and emotional states across groups during the COVID-19 second wave in the Russian Federation. Among students, life quality, especially physical and psychological well-being, was associated with depression and anxiety but was notably independent of stress. Social well-being (including material security, health environment, leisure, and access to medical care) remained stable and unlinked to their emotional state. Among people living with HIV, almost all life quality domains correlated with depression, anxiety, and stress. This group displayed heightened vulnerability to emotional distress, affecting their self-perception, social interactions, and sense of security, thus underscoring the pandemic’s amplified impact on this group’s mental health.

**Disclosure of Interest:**

None Declared

